# Supplementary material for: Are carbon emissions trading and green financial instruments synergistic? -Comprehensive quantitative research based on content analysis
Source: PLoS One. 2024 Mar 7;19(3):e0298601. doi: 10.1371/journal.pone.0298601 (PMC10919601; doi:10.1371/journal.pone.0298601)
Supplement: S1 Data — (ZIP) [file pone.0298601.s001.zip › Data/地级市Z_F工作报告.zip!/╜¡╬≈/╕╙╓▌╩╨/└┤╫╘╣1⁄2╓┌║┼í╛┬φ┐╦╩2╛▌═°í┐_2005.docx]

各位代表：

　　现在，我代表市人民政府向大会作工作报告，请予审议，并请市政协委员和列席人员提出意见。

　　一、2004年的工作回顾

　　2004年是本届政府施政的开局之年。一年来，在中共赣州市委的领导下，市人民政府紧紧依靠全市人民，开拓进取，扎实工作，全面完成了市二届人大一次会议确定的各项任务，国民经济和社会发展取得新成绩。

　　一年来，我们紧紧围绕经济建设中心，抓好发展第一要务，国民经济发展呈现新态势。全市实现生产总值398.01亿元，增长12.7%，增幅比上年提高0.2个百分点，为1997年来的最高增速；财政总收入38.35亿元，增长22.2%，其中地方财政收入突破20亿元，达到22.84亿元,增长16.1%，是近几年来增速最快的一年。

　　农业经济全面发展。积极贯彻执行中央和省一系列惠农政策，农业税降低3个百分点，为农民减负1.22亿元；粮食直补和良种补贴1.41亿元；“三农”支出5.05亿元，增长23.7%。实现农业增加值114亿元，增长4.2%。粮食作物播面768.15万亩，比上年增加141.44万亩，增长22.6%；粮食总产257.39万吨，增长29.3%。农业结构得到优化，果业、商品蔬菜、草食畜禽和特种水产、苗木花卉等主导产业进一步发展壮大，以脐橙为主的水果产量45.5万吨，增长31.63%。农业产业化稳步推进，各类合作经济组织1600多个，年产值100万元以上的农业龙头企业196个，比上年增加41个，其中1000万元以上的102个，增加34个；龙头企业带动农民50多万户。

　　工业经济提速增效。规模以上工业企业实现增加值45.32亿元，增长39.64%；销售收入168.58亿元，增长52.06%；利税18.96亿元，增长45%，超额完成省政府提出的工业“三年翻番”目标任务。工业对全市经济增长的贡献率达到42%，比上年提高8.4个百分点。六大主导产业的地位继续提升，实现销售收入、利税分别占规模以上工业的74.9%和78.5%。50户重点企业支撑作用增强，实现销售收入和利税分别占规模以上工业的51%和68.4%。销售收入过亿元的企业40户，利税超千万元的企业33户。工业园区发展良好，全市16个工业园区实际到位资金96.13亿元，入园企业1176个，实现销售收入109.62亿元，增长55.16%；实交税金6.14亿元，增长120%；安置就业21.34万人，增长59%。

　　商贸服务业加速发展。第三产业实现增加值151.22亿元，增长14.5%；社会消费品零售总额134.91亿元，增长12.3%。市场体系建设不断加强；城市社区服务功能进一步完善。旅游业在经济发展中的地位不断上升，全年共接待国内外游客591.3万人次，旅游总收入34.74亿元，增长17.36%。金融业稳步发展，全市金融机构年末存、贷款余额达到430.65亿元和242.82亿元，分别增长16.30%和2.72%。房地产和通信服务业继续发展，交通运输业呈现新局面。

　　一年来，我们紧紧围绕增强发展活力，全面实施“对接长珠闽，建设新赣州”发展战略，改革开放迈出新步伐。“十大体系”建设进展顺利。完成了体系设计、方案编制、宣传、培训等工作，进入了全面实施阶段，配套文件正在陆续出台。市行政服务中心、陆路口岸、公共保税仓库等一批“十大体系”硬件建设项目已开工，有的已竣工运行并开始发挥作用。

　　各项改革稳步推进。13个部门进行了机构改革和职能调整，建立了国有资产监管新体制，国资委正式挂牌运作。调整交通公路管理职能，交通体制改革取得重大突破。组建了城市规划建设管理新机构。对部分县（市）下放了经济管理权限。事业单位改革进一步深化，以聘用制、人事代理制为主的人事制度改革和分配制度改革全面展开，有1945个事业单位实行了全员聘用制，占事业单位总数的48.7%。农村改革步伐加快，土地流转稳步进行，农税改革及各项配套改革继续深化，“一事一议”负担卡和专用收据制度全面实行，80%以上的乡镇实现了村级财务“村有乡管”。财政、投融资、行政审批、粮食流通等改革取得积极进展。

　　所有制结构进一步优化。个私经济健康快速发展，个私企业12.32万户，从业人员46.01万人，注册资本115.61亿元，分别增长3.18%、10.65%和28.68%。个私经济实现工商税收13.94亿元，增长37.09%；非公有制经济占经济总量的比重比上年提高3个百分点。

　　开放型经济形势喜人。新批外资项目208个，实际使用外资5.39亿美元；引进内资项目1055个，实际引进内资176.11亿元。对外贸易快速增长，有自营出口经营权的企业156家，比上年增加47家；外贸进出口总额4.37亿美元，增长89%，其中出口2.89亿美元，增长122%。

　　一年来，我们紧紧围绕改善发展环境，加强基础设施和城市建设，城乡面貌发生新变化。基础设施建设投入继续增加。完成全社会固定资产投资158亿元，增长1.6%，基本建设投资74.97亿元。17个市属重点工程项目已开工建设，赣定高速公路、赣丰线赣州至崇义段建成通车，赣龙铁路全线贯通，三康庙至橡胶坝防洪工程已完工。建成瑞金至潭东和万安至潭东220千伏输变电线路工程，220千伏嘉定变电站投入使用。机场迁建、瑞金（赣州）电厂、赣韶高速公路、昆厦高速公路瑞金段、赣州火车站扩建等重大项目的前期工作进展顺利。数字赣州、吉安至赣州500千伏输变电工程抓紧实施。全年完成县、乡、村公路改造1924公里，实现90%以上乡镇通水泥路、83%以上行政村通公路、100%的乡镇和68%的行政村通客运班车。水利建设投资2.6亿元，对88座病险水库进行了除险加固，新建和改造了一批水利设施，新增有效灌溉面积3.9万亩、旱涝保收面积5.46万亩。

　　城乡一体化进程加快。城市化率比上年提高1.67个百分点，达到28.52%。中心城区投资2500万元，初步完成了“三江六岸”城市设计和赣州市总体发展规划；投资2.5亿元，对红旗大道、古城墙等城市基础设施进行了大规模改造和“三位一体”整饰。中心城区建成区面积扩大至38.8平方公里，城市面貌发生了新变化。全市城市建设投入加大，新建改建城市道路面积239万平方米，新增城市供水能力6.2万吨/日，新增住宅面积146.43万平方米，新增人均公共绿地面积0.61平方米，建成区绿地率为27%，城市建成区面积扩大至173平方公里。“五新一好”新农村建设全面展开，“三清三改”取得阶段性成果。

　　一年来，我们紧紧围绕关注民生，切实解决关系群众利益的实际问题，人民生活水平有新提高。城市居民人均可支配收入7388元，增长9.88%；农村居民人均纯收入2553元，比上年增加313元，增长13.97%；全市居民消费价格水平102.8%。就业和再就业工作得到加强，全市新增城镇就业5.26万人，“4050”人员实现再就业7240人，城镇登记失业率3.4%，有3.4万国有企业下岗职工实现再就业，国有企业下岗职工减至4963人，公民职业化培训12.44万人，其中9.26万人实现就业再就业。“两个确保”、“一个低保”得到巩固提高，33.44万人参加基本医疗保险，29.5万职工参加失业保险，养老保险参保人员24.02万人，全市10.51万人享受城市最低生活保障。中心城区廉租房建设完成第一期工程9.8万平方米。在全省率先对农村贫困群众及重点优抚对象实行农业税减免，减免金额915万元。农村特困群众社会救助制度进一步完善，救灾救济工作扎实有效,优抚政策落实到位。完成深山区、库区贫困群众移民搬迁1.2万人。

　　一年来，我们紧紧围绕促进社会全面进步，大力发展各项社会事业，三个文明建设取得新进展。各项社会事业协调发展。科技创新能力增强，全市通过科技成果鉴定28项，评定科技进步奖25项，获省科技奖4项，申请专利71项。“重要技术标准研究试点城市”项目开始启动，“可持续发展试验区”项目取得实质性进展。教育事业稳步发展，农村义务教育得到巩固提高，高中阶段教育规模继续扩大，初中升高中比例达到55%，普通高中在校生12.28万人，比上年增加1.36万人。教育结构布局调整进一步完善，教育质量和办学效益有新的提高。以国家公务员、专业技术人才、企业经营管理人才为主体的人才队伍建设得到加强。文化广播电视卫生体育等社会事业全面发展，文艺创作和演出活动丰富多彩，获奖项目增多。广播和电视人口覆盖率分别达92.6%、93.8%。市、县两级突发公共卫生事件应急指挥机制和救治体系进一步健全，全省设区市第一座疾控中心大楼建成投入使用，新型农村合作医疗试点工作稳步进行。食品药品安全专项整治行动广泛开展。群众性体育活动蓬勃发展，竞技体育取得较好成绩。坚持不懈地做好人口和计划生育工作，全面完成计划生育的各项任务，全市计划生育率92.33%，人口自然增长率8.17‰。土地市场秩序整顿取得成效，顺利通过国务院的检查验收。国防动员与后备力量建设得到加强。生态环境进一步改善。拥军优属、优抚安置、监察、审计、物价、统计、人防、水文、气象、防震减灾、对台、民族宗教、外事侨务、新闻出版、地方志、档案、老龄、妇女儿童等各项工作都取得了新的成绩。

　　精神文明和民主法制建设继续加强。城乡文明水平和公民思想道德水平进一步提高，文明社区、文明村镇、文明行业建设活动广泛深入，未成年人思想道德教育扎实推进。世界客属第十九届恳亲大会暨首届客家文化节的成功举办，确立了赣州的客家摇篮地位，扩大了对外交流，提高了赣州的知名度。群众性自治组织蓬勃发展，新农村建设理事会、农民道德评议会、妇女禁赌会、“五老”宣讲队等群众自治组织达3200多个。以文明示范窗口为重点，广泛开展了“共铸诚信”活动。民主法制建设进一步加强，办理人大代表建议和政协提案的质量有较大提高，与各民主党派、工商联和人民团体的联系更加密切。政府职能转变步伐加快，认真修订了《市政府工作规则》，完善政府及其部门规范性文件制定和备案工作程序规定，有效提高了政府决策科学化、民主化水平。普法教育、行政执法和行政复议工作得到加强。强化社会治安综合治理，社会秩序保持良好，人民群众对社会治安的满意度提高。强化安全生产，事故起数、死亡人数分别下降40.5%和18.7%。信访工作领导责任制进一步完善，各类矛盾和不稳定因素不断化解。廉政建设和反腐败斗争深入开展，社会政治稳定局面得到进一步巩固。

　　各位代表，过去一年取得的成绩，是全市人民齐心协力、艰苦奋斗的结果。在此，我谨代表市人民政府，向全市工人、农民、知识分子和干部，向所有社会主义劳动者和建设者，向驻市人民解放军、武警官兵、公安民警，向各民主党派、工商联、人民团体和各界人士，向所有关心支持赣州现代化建设事业的海内外朋友，表示衷心的感谢并致以崇高的敬意！

　　回顾过去的一年，取得的成绩令人鼓舞，但我们也清醒地看到经济社会发展中存在的矛盾和问题。一是经济总量小，发展速度慢。生产总值、财政收入等主要经济指标的绝对值偏小，人均值低于全省和全国平均水平，经济增长速度与周边特别是沿海发达地区相比差距较大。二是基础条件差，投资规模小。能源、交通等基本建设欠账多，基础设施仍较落后，多元化的投资格局尚未形成，投入不足、投资增幅小，与全省全国高速增长形成强烈反差。三是结构不合理，产业层次低。三次产业中二、三产业特别是二产比重偏低。产业规模小，支柱产业不明显，资源优势尚未充分发挥，经济发展的效益较差。四是经济社会发展的深层次矛盾还较突出，政府自身建设有待加强。适应市场经济的体制机制尚未完全建立起来。农民增收和城乡就业压力大，财政增收困难，支出缺口大，社会保障形势严峻，部分群众生活比较困难，影响社会稳定的因素仍然存在。一些部门不能依法行政和办事，少数工作人员不廉洁的现象还时有发生。这些问题需要我们认真及时地加以解决。

　　二、2005年经济社会发展的主要任务和需要把握的要点

　　2005年是“十五”计划的最后一年，也是我市实施“对接长珠闽，建设新赣州”发展战略的重要一年。从全球来看，世界经济将继续走强，全球经贸合作、产业转移和跨国投资的步伐加快，有利于我们扩大对外开放，提高利用外资的规模和水平。从国内来看，区域经济一体化加快推进，泛珠三角经济圈联系日益紧密，有利于我们更多地承接沿海地区特别是珠三角的产业转移。从市内来看，随着基础设施的逐渐完善和体制机制的不断创新，许多优势资源将得到更好地开发利用，发展壮大支柱产业具有良好条件。但前进道路上也会遇到不少困难和问题，经济生活中不健康不稳定因素尚未根本消除。我们一定要认清形势，保持清醒头脑，牢牢把握好重要战略机遇期，增强加快发展的责任感和紧迫感。

　　今年政府工作的总体要求是：以邓小平理论和“三个代表”重要思想为指导，认真贯彻党的十六届三中、四中全会和中央经济工作会议、省委十一届八次全体（扩大）会议精神，按照省委把赣州建设成为江西南部区域经济中心城市和对接珠三角的“桥头堡”的要求，紧紧围绕市第二次党代会确定的目标任务，全面落实科学发展观，把握机遇、调整结构，深化改革、扩大开放，统筹兼顾、突出重点，以人为本、协调发展，深入推进“对接长珠闽，建设新赣州”发展战略，优化发展环境，提升发展水平，增强发展后劲，加速工业化、农业产业化和城市化进程，在构建和谐社会中实现经济社会全面协调可持续发展。

　　今年全市经济社会发展的主要预期目标是：生产总值增长12.5%，财政总收入增长13%，全社会固定资产投资增长18%，社会消费品零售总额增长13%，居民消费价格总水平控制在104%以内，实际利用外资增长20%，5000万元以上的工业项目投资完成100亿元，城镇居民人均可支配收入增长5%，农民人均纯收入增长8至10%，城镇登记失业率控制在4.5%以内，人口自然增长率控制在8.2‰以内。

　　完成好今年的目标任务，必须始终坚持用科学发展观统领经济社会发展全局，切实转变经济增长的内涵和资源利用的方式，不断提升产业层次和经济发展水平，加快构建和谐社会。重点必须把握以下几点。

　　突出第一要务，着力抢抓发展机遇。始终把加快发展作为第一要务，营造聚精会神搞建设，一心一意谋发展的浓厚氛围。树立更加强烈的责任意识和机遇意识，不断丰富把握机遇的战略思维，增强抓住机遇的胆识和魄力，提高用好机遇的能力和水平。注重经济结构优化和综合竞争力提升，实现在抢抓机遇中增创优势，在应对挑战中争取主动，在超越自我中寻求突破，开创发展的新局面。

　　突出改革创新，着力增强内在发展动力。以在全省率先完善社会主义市场经济体制为目标，以建立和完善“十大体系”为总抓手，进一步推进体制机制创新，力争在重点领域和关键环节实现新的突破，努力形成有利于促进经济社会全面协调可持续发展的长效机制。大力培育创业、和谐的社会文化，激活创业细胞，激发创业激情，弘扬创业精神，营造“创业富民、和谐兴市”的文化氛围，让一切创造社会财富的源泉充分涌流。

　　突出外向带动，着力推进大开放主战略。充分利用国际国内两种资源、两个市场，从更大范围、更广领域、更高层次上扩大开放。发挥我市资源、区位等比较优势，主动参与“长珠闽”特别是珠三角经济圈的分工协作，拓展获得技术、人才、资本等要素的空间，加快与珠三角的“接轨”与“融入”，逐步实现产业互补、市场互通、资源互用、体制互融、政策互联，努力把赣州建设成为江西南部的区域经济中心城市和对接珠三角的“桥头堡”。

　　突出统筹兼顾，着力推动全面发展。把发展县域经济作为统筹城乡发展的重大举措，扩大县（市、区）经济管理权限，从政策措施和生产要素等方面加大支持力度，增强县域自主发展能力，壮大县域经济实力。坚持以工业化为主导、以城市化为支撑、以农业产业化为基础，创新机制，协调推进“三化”进程。大力培育支柱产业，促进产业规模扩张和优化升级。统筹经济社会发展，坚持科教兴市、人才强市，加大社会事业改革和投入力度。注重人与自然和谐发展，加强生态建设和环境保护。

　　突出执政为民，着力维护群众利益。牢固树立正确的政绩观和群众利益无小事的思想，把群众呼声和意愿作为指导工作的第一信号，想问题、办事情、作决策，一切为人民群众着想；把关心和服务群众作为第一职责，为群众诚心诚意办实事，尽心尽力解难事，坚持不懈做好事；把致富和造福群众作为第一追求，改善生活质量，增强健康素质，努力提高人民生活水平；把人民群众是否满意作为检验和衡量政府工作的第一标准，不盲目攀比，不急功近利，不做表面文章，不搞华而不实的政绩工程。把握好改革的力度、发展的速度和社会可承受的程度，切实维护城乡社会的稳定与祥和。

　　三、千方百计加大投入，扩大经济发展规模

　　加大招商引资力度，提高经济外向度。精心组织，强势推进，掀起上下联动、政企互动的招商引资新热潮。创新招商引资方式，走专业招商、中介招商、产业招商、网上招商、以商招商的新路子。突出抓好工业六大主导产业的招商引资，充分利用现有产业资源和产业优势招商，加快产业聚集和产业链延伸。不断拓展招商领域，扩大农业、基础设施、社会事业和服务领域的引资份额，提升招商引资的规模和水平。加强对重点国家和地区、重点企业和项目的招商力度，实施重大项目招商年工程，组织好有影响力的大型招商活动，想方设法引进一批技术含量高、发展前景好、互补优势强的重大项目。增加招商小分队，明确各驻外机构的招商目标，加大对招商引资的考核力度。建立健全重大项目的快速反应和跟踪服务机制，切实帮助企业解决用地难、招工难等问题。改善外贸通关条件，扩大我市农产品和加工贸易的出口规模。加强与“长珠闽”特别是珠三角的经济合作与交流，做好对外工程承包和劳务输出工作。

　　加强项目建设，争取国家投入。推进项目带动战略，突出抓好以交通、能源为主的重点项目建设，赣州机场迁建、昆厦高速瑞金段、赣韶高速公路、赣州火车站扩建、县城电网改造项目争取年内开工建设；赣龙铁路、500千伏输电线路及其配套的220千伏输电线路投入使用；完成瑞金（赣州）电厂的前期准备工作，协助配合赣韶铁路年内立项。启动红色旅游、社区服务等工程。完成国省道200公里、农村公路1200公里改造任务。精心组织项目研究与策划，以工业项目和今年国债重点支持的“五大投资领域”项目为主，争取开发新进入市级储备的项目600个，投资总额200亿元以上。加强在建项目的管理，保证项目质量与进度。做好“十一五”规划的编制工作。

　　加快城市建设，扩大投入载体。高度重视规划修编和实施管理，突出规划的先导作用。以产业配套为重要纽带，增强中心城区的集聚能力和辐射带动能力，加速形成中心城区为核心的一小时经济圈。加快城市基础设施、公用事业建设步伐，完善城市功能和扩大城市容量。中心城区完成翠微路三期、京九二路南段、黄金三路西段建设。认真做好赣江大桥、章江大桥和游泳馆、射击馆、博物馆建设的前期准备工作，加快青少年活动中心、市接待中心、市政中心北区、市传染病医院、“123”工程、污水处理、红旗大道延伸、宋城公园完善、廉租房和经济适用房及三中新校区等工程的建设。继续进行老城区主次干道和小街小巷整治，加强历史文化名城保护和历史街区修整。围绕创建国家园林城市的目标，着力提高中心城区综合管理水平。抓好瑞金、龙南两个次中心城市、14个国家重点镇、22个省级重点镇建设。提高城市综合资源经营水平，推进土地一级市场垄断和计划收储、供应工作，开放城市基础设施和公用事业投资市场。

　　激活民间投资，拓宽投资渠道。深化投融资体制改革，加快建立和完善投资主体多元化、资金来源多渠道、投资方式多样化、项目建设市场化的投资格局，确立企业的投资主体地位，激活民间投资，鼓励社会投资。进一步放宽民间资本进入基础设施、公用事业等法律法规未禁止的领域，支持民间投资与政府投资合作发展。选择一批市政、环保、公路、文化教育等基础设施和经营性的公益事业项目向社会公开推介，鼓励社会资本以独资、合资、合作、联营、特许经营、项目融资等方式参与建设和经营。

　　四、调整和优化经济结构，提高经济增长质量和效益

　　加大产业培育力度，促进工业跨越式发展。坚定不移地实施工业立市方略，加快新型工业化步伐，实现全年规模以上工业增长高于全市GDP增速8个百分点、高于全省工业增速4个百分点的目标。围绕做大六大主导产业，加快建设卷烟、酿酒、制药、造纸、制鞋、服装纺织、新型干法水泥7大产品全省重要生产基地，稀土、钨、锡、钴金属及材料、汽车变速箱和氟、盐化工6大产品全国重要生产基地。加大产业整合力度，合理布局分工，向外引大联强，发展深加工，力争用5年左右时间，把稀土、钨和氟、盐化工培植成三个销售收入超百亿元的产业。完善扶优扶强措施，集中力量做强50户左右的重点企业，引导企业加强战略规划研究和资本运作，完善发展动力、管理创新、技术创新三大机制，培育自主知识产权产品和知名品牌，谋求与世界500强和国内200强企业合作，积极创造条件上市融资。争取3户企业销售收入过10亿元或利税过亿元，三年内有8至10户企业销售收入过10亿元或利税过亿元。加快德国格特拉克公司投资江铃齿轮生产汽车变速器、钨锡铜深加工基地和氟、盐化工产业基地等15个重大项目的建设。整合提升各类工业园区，完善园区基础设施建设，提高投资密度、产出水平，推动企业集聚、项目集中和土地集约化利用。培育一批特色工业园区，引导上下游产品和配套产品集中，促进资本和产业集聚。

　　以农民增收为核心，加快农业和农村经济发展。坚持“多予、少取、放活”的方针，认真贯彻中央一号文件精神，坚决落实免征农业税政策，已经实行的优惠政策不变，已经给的实惠不减，并且还要加大政策扶持。稳定粮食播种面积，提高粮食综合生产能力。加大产业结构调整力度，全面完成农业主导产业发展规划和区域特色优势产业发展规划，优化产业区域布局，加快优势农产品产业带建设，发展壮大一批过亿元的龙头企业。做大做强果业、商品蔬菜、苗木花卉、生猪四大主导产业，扶持发展烟叶、特种水产、工业原料林、毛竹、鸡鸭和草食畜禽等若干个区域特色优势产业，经过若干年的努力，争取把脐橙发展成为百亿元的产业。加快农村土地流转，尽快建立与完善农村各类专业、合作经济组织。抓好农业产业化经营体系、农业科技创新与应用推广体系、农产品质量安全保障体系、农产品市场与信息服务体系建设。加强赣南脐橙原产地域保护，实施赣南脐橙品牌建设工程。实施科技兴农战略，加强农民先进实用技术和职业技能培训，大力推广新品种、新技术。推进农民知识化工程，加快农村劳动力转移步伐。抓好扶贫开发工作，完成1.4万人库区、深山区移民扶贫搬迁任务。加大农田水利基本建设力度，完成75座病险水库除险加固。

　　推进新农村建设，促进城乡共同繁荣。推进“五新一好”新农村建设，年内完成全市所有铁路、国省道等公路沿线行政村以及1000人以上村庄的规划编制。各级各部门要相互配合，搞好服务，形成合力，引导参与农村全面展开“三清”，扎实推进“三改”，整治农村环境，美化净化村容镇貌。组织资源、技术和项目资金，加快农村新兴产业发展。市、县两级财政预算要安排一定比例的专项资金，并整合各类涉农资金支持新农村建设。充分调动和发挥村民自治组织的积极性，提高农村公共事务管理与服务水平。

　　以发展现代服务业为重点，提升第三产业发展层次。把旅游业作为赣州经济发展的重要产业来抓，加大旅游资源规划、开发、整合力度，搞好“中国优秀旅游城市”和“红色故都、客家摇篮、江南宋城、三江源头”四大旅游品牌的宣传推介，抓住国家实施红色旅游"121"工程的机会，搞好红色旅游开发和建设，打造知名景区和精品线路。积极促进区域旅游合作，实现资源共享利益双赢。加快地方金融机构改革步伐，积极引进国内外金融机构，做大市商业银行，加大农村小额贷款力度，提升金融服务水平。推进电子商务等信息技术的开发利用，打造现代化“信息高速公路”，加快信息服务业发展。改造提升传统商贸业，支持国内外知名的大型商贸零售企业到赣州落户。整合交通、港口、邮政等物流资源，加快现代物流业发展。搞活住房二级市场，有序推进房地产开发，促进房地产业健康发展。积极扶持医疗、家政、幼托、养老、维修、文化、休闲等各类社区服务业，培育会计、审计、公证、评估、鉴定、拍卖等中介服务业和法律服务业，建设城乡一体化服务网络。

　　五、适应市场化进程，不断创新体制机制

　　抓紧实施“十大体系”。加强“十大体系”建设的组织领导，健全市、县两级工作网络，抓紧有关配套文件的制定，促进各项配套政策措施尽早出台，及时做好政策落实中的督查、反馈和评估工作。按照“成熟一个运作一个”的要求，着力推进政策法规、金融服务、口岸服务、人力资源、信息网络、社会保障、物流等体系建设先行突破。抓紧抓好综合示范县和单项示范县的试点示范工作，通过示范带动推进全市“十大体系”建设。按照体制机制对接和创新的要求，整合相关部门的职能和机构。进一步抓好“十大体系”的基础设施建设，推进硬件、软件建设协调发展。

　　稳步推进各项改革。完善国有资产管理体制，深化行政事业性国有资产管理体制改革，构建新的国有资产经营平台，加快国有资产的资本化运营，做大、做强、做优、做活国有资产。抓好市、县级产权交易机构建设，建立健全土地、房产和企业交易市场，促进各类产权合理流转。深化财政体制改革，加强部门预算管理，完善国库集中支付和政府采购制度，加快构建公共财政体系；坚持依法理财，强化专项资金管理，提高财政资金使用效益。推进事业单位改革，对行政性事业单位，进行清理和规范；对公益性事业单位，抓好布局调整和结构优化；对经营性事业单位，按照市场化、社会化、产业化方向加快改制转企步伐。创新区域经济协调发展机制，逐步实现区域间资源、信息、人才共享，产业融合，避免无序竞争。深化粮食流通、林业产权、交通管理、新闻出版和乡镇管理等方面的改革，增强经济发展动力。

　　加快非公有制经济发展。继续引导和鼓励非公有制经济参与国有、集体企业改制和资产重组，进一步放宽非公有制企业的市场准入，认真落实鼓励非公有制经济发展的政策措施，在税收政策、规费标准、金融扶持、政府服务等方面享受国民待遇，使非公有制经济更广泛地进入三大产业。积极组织对个私业主的学习、培训、考察活动，帮助个私业主提高素质，创新管理，建立现代企业制度。

　　六、大力发展各项社会事业，进一步推动经济社会全面协调发展

　　继续推进科教兴市、人才强市战略。深化科技管理体制改革，加强产学研联合，积极推动科技成果向现实生产力转化。加快建立和完善以企业为主体的技术创新体系，鼓励企业进行新技术研究和新产品开发。建立多元化科技投入机制，引导民营科技企业及各类科技服务组织加快发展。广泛开展科技交流与合作，争取更多的高新技术项目落户我市。发展教育事业，完善“以县为主”的义务教育管理体制和经费保障机制，整合教育资源，优化中小学网点布局，全面提高“普九”水平。积极发展高中阶段教育和幼儿教育，大力发展职业教育和成人教育，实施公民职业化工程，构筑终身教育体系。鼓励、引导和规范社会力量办学，形成多元化、多渠道投资办学格局。紧紧抓住培养、吸引和用好人才三个环节，加大人力资源开发力度，完善人才政策，推动人才“柔性”流动，建立健全人才资源开发和管理体系，发挥大中专院校在人才强市战略中的优势，集聚更多的人才为赣州的发展服务。

　　大力发展文化卫生体育等社会事业。实施百县千乡文化工程，广泛开展各具特色的广场文化、社区文化、企业文化、村镇文化、家庭文化、校园文化等活动。鼓励和扶持具有浓郁地方特色的文化产品生产，大力弘扬红色文化和客家文化，推动文化艺术繁荣发展。继续推进广播电视“村村通”建设。深化医疗卫生体制改革，加强疾病预防控制体系、医疗救治体系和卫生监督体系建设，全面启动创建卫生村和农村爱国卫生示范镇活动。加强食品药品安全监督，切实保障人民群众的食品和用药安全。积极开展全民健身运动，努力提高竞技体育水平。完善统计制度和方法，认真搞好第一次经济普查。深入开展“双拥共建”活动，加强国防教育和国防后备力量建设。继续做好监察、审计、物价、人防、水文、气象、防震减灾、对台、民族宗教、外事侨务、新闻出版、地方志、档案、老龄、妇女儿童等工作。

　　大力加强精神文明和民主法制建设。认真落实《公民道德建设实施纲要》，加强和改进未成年人思想道德建设，全面实施“留守孩”关爱工程。深入开展群众性精神文明创建活动，组织开展农村创评“文明信用户”活动。按照全省统一部署，广泛开展“建设和谐平安江西，共创富民兴赣大业”的主题教育活动。自觉接受人大的依法监督和政协的民主监督，认真办理人大代表议案、建议和政协提案。广泛听取各民主党派的意见，充分发挥工会、共青团、妇联等人民团体的桥梁纽带作用。健全基层群众自治组织，加强城乡社区建设，完善村（居）务公开和民主管理制度。坚持依法治市，加强法制宣传教育，增强全社会的法律意识和法制观念。积极开展法律援助，加强法律服务。

　　七、坚持以人为本，努力构建和谐平安赣州

　　促进人口、资源、环境协调发展。稳定低生育水平，提高出生人口素质，优化人口结构。落实人口与计划生育目标管理责任制，加强流动人口管理和社会抚养费征管，解决出生人口性别比失调的问题。巩固土地市场秩序整顿成果，严格实行经营性用地招、拍、挂出让制度。进一步规范矿产资源的开采、加工、经营活动，加强对稀土、钨等保护性矿种的规范化管理。实施天然阔叶林保护工程，抓好林政管理。推行清洁生产，发展循环经济，推进节约型社会建设。强化城市环境综合治理，加大对工业污染源、大气和水污染、噪声的整治力度，逐步推行绿色生产、绿色消费。加强赣江、东江源头区域生态保护。

　　做好就业、再就业和社会保障工作。落实就业再就业政策，把扩大就业和提高就业质量放在经济社会发展更加突出的位置。大力发展就业容量大的产业、劳动密集型企业，积极创造和增加就业岗位，鼓励和扶持下岗失业人员灵活就业和自主创业。加快建设全市统一、规范的劳动力市场，完善公共就业服务机构和街道社区劳动保障平台功能，加强就业培训和创业培训，促进下岗失业人员实现再就业。巩固“两个确保”成果，加强“三条保障线”的衔接，基本完成国企下岗职工基本生活保障向失业保险的并轨工作。完善城镇职工基本养老保险制度，深化基本医疗保险制度改革，逐步建立新型农村合作医疗制度，健全失业保险制度，加强社会保险基金征缴，依法推进各类所有制企业、个体工商户和进城务工农村劳动者的扩面征缴工作，确保符合低保条件的城市居民应保尽保。扎实做好农村特困户救助工作，保障农村“五保户”的合法权益，“五保户”集中供养率达到60%以上。

　　全力维护社会稳定。继续围绕“两杜绝一下降”目标，加强对安全生产工作的领导，抓好制度建设、责任制落实和应急体系建设，增强依法监督力度，预防重特大事故的发生。强化社会治安综合治理，完善社会治安防控体系，依法严厉打击各种刑事犯罪，有效遏制社会丑恶现象，提高人民群众对社会治安的满意度。严格信访工作领导责任制，正确处理人民内部矛盾，及时排查、调处、化解矛盾纠纷，切实解决损害群众利益的问题。

　　八、加强和改进政府工作，提高行政能力和水平

　　在全面推进赣州改革开放和现代化建设事业的进程中，我们面临着繁重而艰巨的任务，各级政府要以“保持共产党员先进性教育活动”为契机，按照科学执政、民主执政、依法执政的要求，在争创一流政务环境和建设群众满意的政府上迈出更大的步伐。

　　转变职能，提高科学管理的能力。围绕经济调节、市场监管、社会管理和公共服务四大职能，创新政府管理模式，变直接管理为间接管理，把该管的管好，不该管的坚决放开。按照市场经济的运行规律和内在要求，主要运用经济和法律手段调节经济。完善社会管理政策和法规，主动围绕公民与企业的需求开展工作，以方便市场主体为导向调整设置工作程序，不断推出新的服务产品和服务方式。继续深入整顿和规范市场经济秩序，建设“信用政府”，提高政府公信力。

　　规范行为，提高依法行政的能力。认真贯彻落实《全面推进依法行政实施纲要》和《行政许可法》，严格按照职权法定、依法行政、有效监督、高效便民的要求，履行职责、办理文件、处理问题、管理事务。深化行政审批制度改革，进一步清理行政审批文件。扩大行政综合执法，切实解决好执法部门职责交叉、多头执法、重复处罚、执法扰民等问题。健全行政执法责任制、评议考核制和执法过错追究制，做好行政复议和行政诉讼工作。

　　以民为本，提高民主行政的能力。树立公民本位的思想，强化民主观念和民主意识，真正做到权为民所用、情为民所系、利为民所谋。健全和完善充分体现民主行政的各项制度，大力推进民主行政的制度化、规范化和程序化，切实保障人民的民主权利。搞好以办事公开和信息公开为主要内容的政务公开工作，健全和完善听证会制度，提高政府机关的行政透明度，保证群众依法行使知情权和参与权。重视民主决策和科学决策，使各项决策更加符合法律、切合市情、适合民意。拓宽和健全监督渠道，把行政权力运行置于有效的制约和监督之下，确保公共行政权力为群众、为社会谋福利。

　　改进作风，提高行政效率。大力实施政府“提速”工程，办文上力求准确及时、简洁精炼，能不发的文尽量不发；办会上力求少开会、开短会，杜绝可有可无的会；办事上力求严格程序、规范运转，坚持谁主管谁负责，实行首问负责制，明确从受理之日起到办结的时限，只要手续齐全、符合规定的，就立即办、马上办。大兴求真务实之风，深入基层调查研究。把各项决策细化、实化为可操作性的办法和措施，把各项任务细化、实化为明确具体的指标，一件一件地落实，一项一项地完成。推行电子政务，运用现代信息技术优化政府组织和管理。完善行政服务中心的运作，提高服务水平。建立和完善目标管理责任制和督查考核责任制，组建行政效能投诉中心，在全市各级机关开展优化发展环境效能监察工作。

　　廉洁从政，提高拒腐防变的能力。坚持教育为先、制度为重、监督为实，抓紧市委惩治和预防腐败体系的落实，把惩防体系分解到每一个部门和单位、每一个岗位和个人，全面建立起制度，并把各级各层次各岗位的制度和责任落到实处。深入开展“行风评议”活动，坚决纠正部门和行业不正之风。所有政府工作人员要牢固树立正确的世界观、人生观和价值观，堂堂正正做人，清清白白从政，干干净净干事，切实做到“不为名所累、不为利所缚、不为欲所惑”，自觉保持“两个务必”，始终坚持廉洁自律、警钟长鸣。

　　各位代表，加快富民强市步伐，是800多万赣州人民的迫切愿望，是历史赋予我们的神圣使命。让我们紧密团结在以胡锦涛同志为总书记的党中央周围，高举邓小平理论和“三个代表”重要思想伟大旗帜，全面贯彻党的十六大和十六届三中、四中全会精神，在中共赣州市委的领导下，坚定信心，振奋精神，与时俱进，真抓实干，为全面完成“十五”计划的各项目标任务而努力奋斗！
